# Implementation and Evaluation of an Alternative Electronic Health Record Tool for Ordering Blood Products in Pediatric Oncology and Stem Cell Transplantation: Mixed Methods Analysis

**DOI:** 10.2196/93346

**Published:** 2026-05-15

**Authors:** Ally Sarna, Aya Finkelstein, Caroline Malcolmson, Renee Potashner, Natalie Pitch, Alessia Forestieri, Marilyn Cooper, Yuquing Feng, Aryan Patel, Tal Schechter, Karim Jessa, Lillian Sung, Adam P Yan

**Affiliations:** 1 Division of Haematology/Oncology, The Hospital for Sick Children, 555 University Avenue, Toronto, ON, M5G 1X8, Canada, 1 4374296924; 2 Department of Pediatric Laboratory Medicine, The Hospital for Sick Children, Toronto, ON, Canada; 3 Department of Pediatrics, The Hospital for Sick Children, Toronto, ON, Canada; 4 Program in Child Health Evaluative Sciences, The Hospital for Sick Children, Peter Gilgan Centre for Research and Learning, Toronto, ON, Canada; 5 Western University, London, ON, Canada; 6 Division of Pediatric Emergency Medicine, The Hospital for Sick Children, Toronto, ON, Canada

**Keywords:** electronic health record, clinical decision support, pediatrics, oncology, transfusion medicine

## Abstract

**Background:**

Repeated blood product ordering is associated with order entry errors and potential patient harm. Traditional electronic health record order sets require repeated re-entry for recurrent transfusions, creating inefficiencies and opportunities for error, and contributing to physician burnout. Historically, we have used order sets to order blood products, which must be re-entered each time a transfusion is needed. Reusable transfusion therapy plans may address these challenges by standardizing and streamlining transfusion workflows. We conducted a pre-post study at a single pediatric academic center, evaluating the implementation of reusable transfusion therapy plans for packed red blood cells and platelets in oncology patients and those undergoing hematopoietic stem cell transplantation.

**Objective:**

The primary outcome was to evaluate the proportion of transfusions originating from the transfusion therapy plans during the postimplementation period. Secondary outcomes included evaluating (1) the proportion of eligible patients with applied transfusion therapy plans, (2) changes in transfusion efficiency (time from laboratory result to transfusion release and administration, premedication timing, and estimated overnight pages), and (3) the impact on safety (guideline-concordant dosing, irradiated product ordering, and transfusion thresholds). We also assessed health care practitioner experience using an adaptation of the technology acceptance model survey.

**Methods:**

The prestudy period consisted of the 1-year preimplementation, and the postperiod consisted of the 1-year post implementation. We used our institution’s enterprise data warehouse (SickKids Enterprise-Wide Data in Azure Repository) to obtain demographic and transfusion details for all eligible patients. The adapted technology acceptance model survey was administered to eligible oncology clinicians.

**Results:**

The preimplementation cohort had 558 unique patients who received a total of 2678 transfusions. The postimplementation cohort had 521 unique patients who received 2777 transfusions. During the postimplementation period, 59% of transfusion orders originated from a therapy plan, increasing to 71% in the final month. Compared with order sets, therapy plan–derived transfusions were released and administered significantly faster following laboratory results (*P*<.001). Guideline-concordant transfusion volumes increased significantly postimplementation for both packed red blood cells and platelets (*P*<.001), as did the ordering of irradiated blood products (*P*<.001). No differences were observed in pretransfusion hemoglobin or platelet thresholds between study periods. Use of therapy plans was associated with an average avoidance of 4 overnight blood product entries per night. Survey responses from nurses and providers demonstrated high perceived usefulness and ease of use, with 95% endorsing continued use.

**Conclusions:**

Reusable transfusion therapy plans improved efficiency, standardized safe ordering practices, and were highly acceptable to clinicians. This longitudinal, noninterruptive electronic health record intervention represents a scalable approach to supporting high-risk transfusion workflows in pediatric oncology.

## Introduction

Blood products, such as platelets and packed red blood cells (pRBCs), are frequently required during pediatric cancer therapy. Ordering blood products is inherently complex, and incorrect orders can lead to severe adverse outcomes for patients [1]. A common cause of blood product safety events is the inappropriate ordering of blood products, including transfusion above recommended thresholds, omission of required premedications, and incorrect dosing [2-6].

Electronic health records (EHRs) can support safer transfusion practices by providing access to comprehensive medication, allergy, and laboratory information [7,8]. Standardized order sets, a grouping of related orders within EHRs, are increasingly used to strengthen workflow efficiency, improve the accuracy and timing of medication administration, and reduce medication errors [9,10]. Ensuring end user evaluation of digital tools to support complex ordering is important to ensure they are achieving the intended impact, are acceptable to end users, and have adequate usability [4,11-13].

A common approach for blood product ordering is the use of an order set, a collection of related orders that are grouped together to facilitate ordering. When patients require repeated transfusions, the order set must be manually re-entered by a health care provider with each transfusion. Given the need for frequent transfusions among patients with pediatric cancer and patients undergoing hematopoietic stem cell transplantation, we identified an opportunity to eliminate the repeated ordering of transfusions using a transfusion therapy plan. A therapy plan differs from an order set in that the orders can be reused longitudinally without a provider needing to manually re-enter them. Transfusion therapy plans allow clinicians to predefine recurring transfusion orders, including indication, volume, precautions, and premedications. We hypothesized that, compared to the traditional order set, a therapy plan would be better for blood product ordering as it might: (1) reduce order entry errors; (2) reduce the time to transfusion; (3) improve transfusion guideline–consistent care; and (4) improve practices consistent with patient-specific needs, such as precautions and premedications.

Therefore, our primary objective was to understand the usage patterns of transfusion therapy plans. Our secondary objectives were to understand how transfusion therapy plans impact: (1) efficiency, (2) safety, and (3) clinician experience.

## Methods

### Study Design and Setting

This study was conducted at The Hospital for Sick Children (SickKids), a large academic pediatric institution in Toronto, Canada. SickKids cares for approximately 350 new patients with cancer and performs 100 hematopoietic stem cell transplants (HSCTs) per year. SickKids has used Epic as its EHR since 2018 [14]. The project consisted of two 1-year periods: November 2023 to October 2024 (preimplementation) and November 2024 to October 2025 (postimplementation) of the transfusion therapy plans. All data were obtained from our institution’s enterprise data warehouse, named SickKids Enterprise-Wide Data in Azure Repository [15].

### Ethical Considerations

This project was approved by the Quality Improvement Review Board at SickKids (QIP-2024-01-26T13-52-53). Informed consent was not required, and participants were not compensated for participation. Patient privacy and confidentiality were maintained through anonymization of data.

### Transfusion Workflows

The workflows for ordering, preparing, and administering blood using both EHR tools (order set and transfusion therapy plan) are detailed in Table S1 in Multimedia Appendix 1. In brief, using the order set for transfusion, orders are entered by a provider at the time a transfusion is needed and cannot be reused, whereas with the therapy plan, the orders can be entered in advance or at the time of transfusion and can be reused. Regardless of how the transfusion orders are entered, a nurse then reviews the orders and “releases” them, rendering them active in the system. One of these orders is a “prepare” order, which specifies the blood product requirements to the blood bank. The blood bank then prepares the requested product and delivers it to the patient’s nurse. The nurse then administers any premedications, uses a “transfuse” order to administer the blood product, monitors for transfusion reactions, and administers the emergency medications if a reaction occurs.

### Transfusion Therapy Plan Creation

Two transfusion therapy plans were created—one for pRBC orders and one for platelet orders. New sections in our “Treatment” activity, where cancer-specific orders are placed, were created for applying the transfusion therapy plans (Multimedia Appendix 2). Both therapy plans contain the prepare and transfuse orders for their respective blood components (MultimediaAppendices 3  4). The blood product orders were configured to maximize adherence to our transfusion volume guidelines. For pRBC orders, if the patient weighs less than 25 kg, providers are presented with order options in mL/kg/dose, and if they weigh 25 kg or more, they are presented with order options in units. For patients less than 25 kg, the maximum mL/kg/dose volume that can be ordered is 10 mL/kg, and for patients more than 25 kg, only 1 unit of pRBC can be ordered at a time. For the platelet orders, if the patient weighs less than 20 kg, providers are presented with order options in mL/kg/dose, and if they weigh 20 kg or more, they are presented with order options in units. For patients less than 20 kg, the maximum dose that can be entered is 10 mL/kg (200 mL), and for patients more than 20 kg, the only dose that can be entered is 1 unit. Hard stops were configured within the orders to prevent ordering transfusion volumes that exceed the recommended maximums. If additional units are required, direct communication with the blood bank is required.

Both plans contain optional premedications to prevent transfusion reactions and emergency medications that can be administered in the case of a transfusion reaction. The pRBC plan also contains a type and screen order. Both plans also include a communication order that indicates in what context the nurse can release the transfusion orders. For the pRBC therapy plan, the default options a provider can select are to transfuse for a hemoglobin level of less than 70, 80, or 90 g/L, and for the platelet therapy plan, the default options a provider can select are to transfuse for a platelet count of 10 or 20 (if febrile), 20, 30, or 50 kcells/µL. However, a provider can manually enter a patient-specific threshold. If a transfusion is needed and the threshold in the communication order is not met, but a transfusion is felt to be clinically indicated by the team (eg, threshold indicates transfuse for hemoglobin of 70 g/dL, and the patient’s hemoglobin is 71 g/dL in a patient with tachycardia and fatigue), an ad hoc one-time nursing communication order can be entered approving the use of the transfusion therapy plan. Dosages of both blood products and supportive care medications automatically update using the patient’s most recent weight. An automated in-basket message to the primary oncology provider is generated every 6 months, indicating that the plan must be reviewed and either renewed or discontinued.

Once the therapy plan is applied and signed, the orders within the therapy plan can be released by a nurse without the need for a provider to enter additional orders. The therapy plan orders can be reused each time a transfusion is required, eliminating the need to reorder blood products each time a transfusion is required.

### Population

Patients with active cancer and those undergoing HSCT were included. Patients with active cancer were defined as those seen in an oncology clinic or admitted to an oncology service with an active chemotherapy treatment plan (defined as 1 chemotherapy day within the last 30 days). Patients with HSCT were defined as any patient within 100 days of the infusion of a cellular therapy product.

### Transfusion Therapy Plan Use

We evaluated therapy plan usage in 3 ways. Our primary outcome was to evaluate the proportion of eligible blood product orders that came from a transfusion therapy plan on a monthly basis. Secondary outcomes included evaluating the proportion of eligible patients who had a transfusion therapy plan applied and the proportion of eligible patients who received a transfusion during the study period and had a transfusion therapy plan applied. Only transfusions given within an oncology or HSCT department were considered (ie, transfusions in the emergency department, operating room, or intensive care unit were not included). The use of the platelet and pRBC therapy plans was tracked separately.

### Impact on Efficiency

We evaluated changes in hospital efficiency in 3 ways. First, for patients who received a blood product, we evaluated the time between the complete blood count (CBC) being reported in Epic and the blood being released and administered by the nurse caring for the patient. These metrics were calculated on a per-transfusion basis. If more than one unit of a blood product was included in a single release, these were counted as a single transfusion, and the release of the transfusion was counted as the time for the first unit. Second, we hypothesized that our transfusion therapy plan might decrease the number of times where a CBC and type and screen were drawn in separate blood draws. We evaluated the proportion of transfusions where the CBC and type and screen were drawn within 5 minutes of each other. Since many inpatients might already have one of these premedications ordered for an alternative indication, we only evaluated premedications for outpatient administrations of blood products. Finally, we evaluated the estimated change in overnight transfusion ordering related to eliminating the need for providers to order blood overnight. We calculated the number of blood product orders placed between 7 PM and 7 AM.

### Impact on Safety

To evaluate changes in blood product ordering patterns, we evaluated the mean pretransfusion hemoglobin and platelet levels prior to the transfusion of blood or platelets, respectively. All oncology patients and patients with bone marrow transplants (BMTs) should have their blood ordered as irradiated. We evaluated the proportion of blood products ordered with this precaution during both periods as well. We also evaluated the proportion of patients receiving an appropriate transfusion volume. For blood, the maximum appropriate transfusion volume was defined as 10 mL/kg for patients weighing less than 25 kg and 1 unit for patients weighing more than 25 kg. For platelets, the maximum appropriate transfusion volume was defined as 10 mL/kg up to 200 mL for patients weighing less than 20 kg and 1 unit for patients weighing more than 20 kg. These volumes are in accordance with our internal blood transfusion policy. In the postimplementation period, we also compared the same safety metrics for transfusions that came from a therapy plan compared to those that came from an order set. In addition, we hypothesized that premedications would be less frequently missed with the use of the new therapy plan. We calculated the proportion of patients who had an allergy to blood or platelets listed in Epic and who had a premedication (hydrocortisone, diphenhydramine, cetirizine, or acetaminophen) ordered within 5 minutes of the blood product they were allergic to being ordered.

### Health Care Practitioner Experiences

To assess health care practitioner experiences using the transfusion therapy plan, a survey was administered 6-months post go-live of the transfusion therapy plan to physicians and nurses who care for oncology patients and those with BMT via REDCap (Research Electronic Data Capture; Vanderbilt University). The survey consisted of three parts: (1) demographic information about the respondent, (2) an abbreviated version of the technology acceptance model (TAM) questionnaire, and (3) specific questions related to the impact on transfusion-related practices [16-18]. Demographic information included health care role (oncology attendings, oncology fellows, pediatric residents, clinic nurses, or ward nurses), primary area of practice (leukemia or lymphoma, solid tumor, neuro-oncology, or BMT), and duration of employment at our institution. The TAM is a questionnaire that assesses technology acceptance across two main constructs: (1) perceived usefulness of the technology and (2) perceived ease of use of the technology. The TAM has been shown to be both reliable and valid. To assess the impact of the transfusion therapy plan on specific blood ordering practices, novel questions were developed by a subset of the study team with expertise in questionnaire development, clinical informatics, usability, and transfusion medicine.

### Statistical Analysis

Descriptive statistics were used to summarize demographic data, transfusion history, and therapy plan usage. To determine factors associated with the use of a therapy plan and differences in outcomes between order set and therapy plan–derived transfusions, the chi-square test and Fisher exact test were used for categorical variables, and the Wilcoxon rank-sum test was used for continuous, nonnormally distributed variables (RStudio version 1.4.1717; Posit PBC).

## Results

### Demographics

The demographics of the preimplementation and postimplementation cohorts are shown in Table 1. The preimplementation cohort included 558 patients and the postimplementation cohort included 521 patients. No statistical differences in the baseline characteristics of the 2 cohorts were identified.

**Table 1.** Cohort characteristics of patients who received at least 1 transfusion.

Characteristic	Preimplementation cohort (n=558)	Postimplementation cohort (n=521)	*P* value
Sex, n (%)			.62
Male	319 (57)	290 (56)	
Female	239 (43)	231 (44)	
Age (y), median (range)	7.7 (0-24)	7.8 (0-20)	.80
Disease group, n (%)			.23
Bone marrow transplant	132 (24)	95 (18)	
Leukemia or lymphoma	338 (61)	307 (59)	
Neuro-oncology	70 (13)	75 (14)	
Solid tumor	109 (19.5)	107 (21)	
Inpatient admission during period, n (%)	374 (67)	351 (67)	.90
Outpatient clinic visit during period, n (%)	499 (89)	474 (91)	.39

### Transfusion Therapy Plan Use

**Figure 1.**
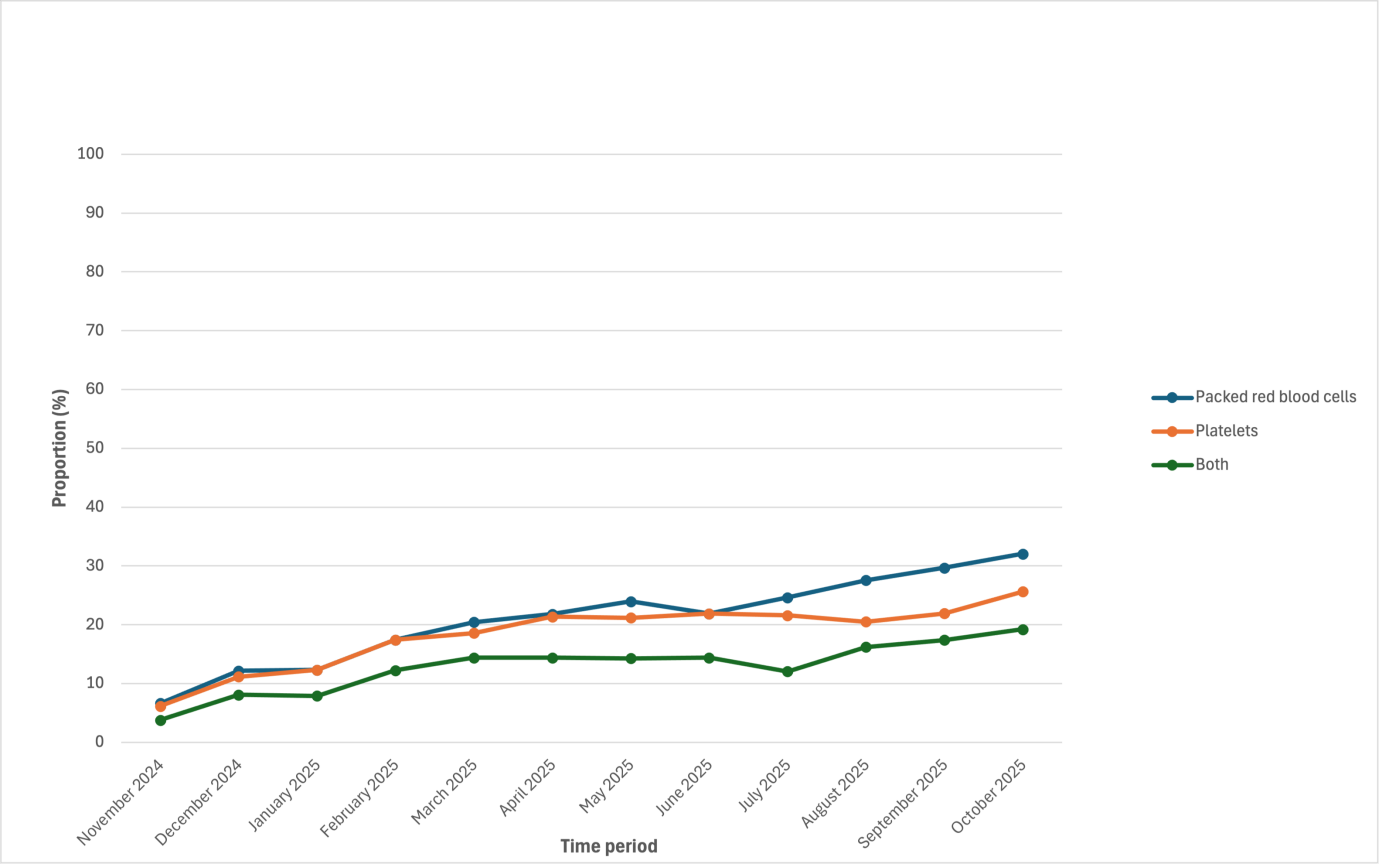
Changes in the usage of transfusion therapy plans over time—proportion of eligible patients with a transfusion therapy plan.

During the 1-year postimplementation period, 26% (n=138) and 24% (n=125) of eligible patients had a pRBC or platelet therapy plan applied, respectively. Of the 238 patients with either transfusion therapy plan applied, 59% (n=141) had both plans applied. The proportion of eligible patients who had either transfusion therapy plan applied ranged from 32% (97/307) for patients with leukemia or lymphoma to 100% for patients with neuro-oncology (75/75) and BMT (95/95; Table S2 in Multimedia Appendix 1). Of the 220 patients who received pRBCs, 58% (n=127) had a pRBC transfusion plan, and of the 158 patients who received platelets, 67% (n=106) had a platelet transfusion plan. Overall, during the postimplementation period, 59% (n=1634) of transfusion orders originated from a transfusion therapy plan. The proportion of eligible patients with either therapy plan, the proportion of patients who received a transfusion who had a therapy plan, and the proportion of transfusion orders coming from a therapy plan, stratified by month, are shown in Figures1^3^. In the last month of our evaluation period (October 2025), 39% (60/156) of eligible patients had at least 1 therapy plan, 60% (35/58) of patients who were transfused had the corresponding transfusion therapy plan, and 72% (88/122) of all transfusion orders came from a therapy plan.

**Figure 2.**
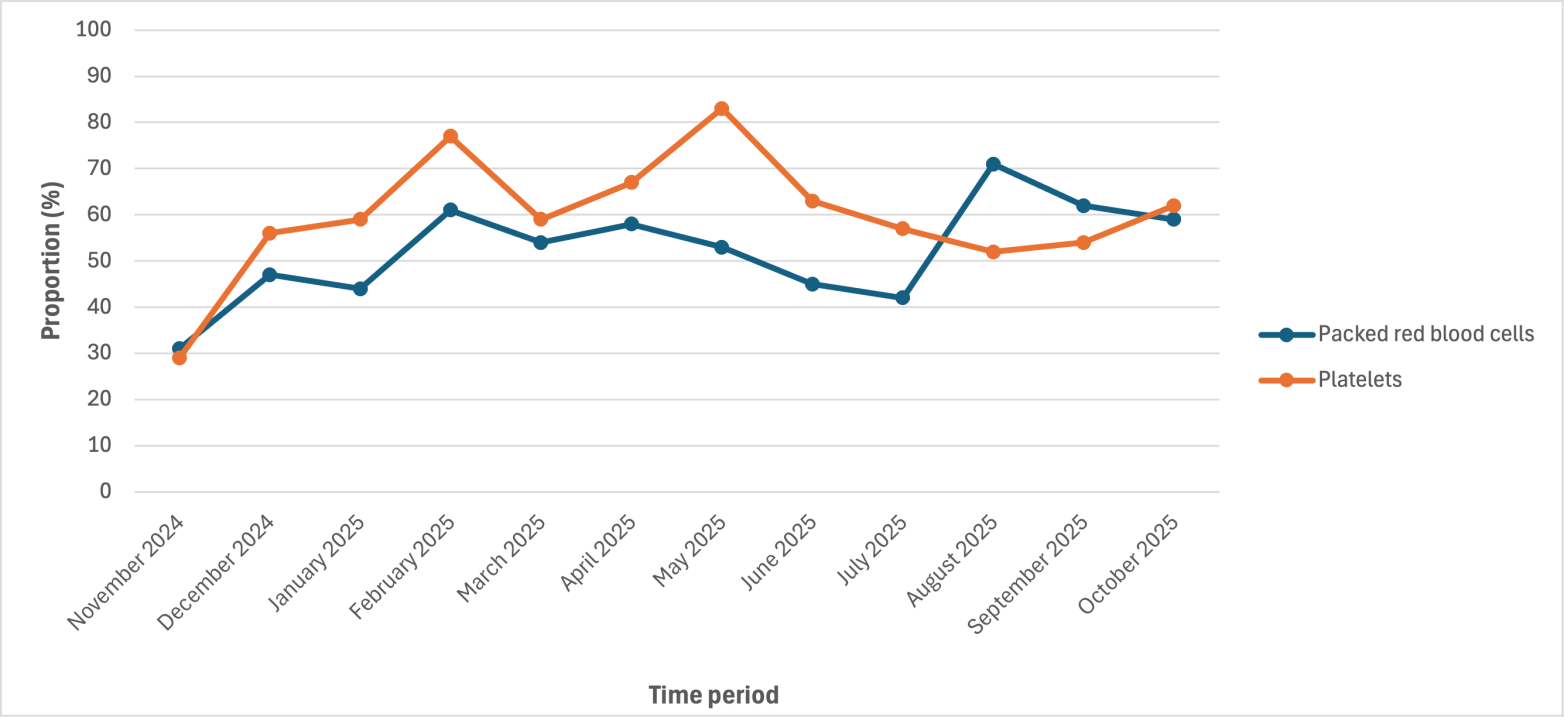
Proportion of transfused patients with the corresponding transfusion plan applied.

**Figure 3.**
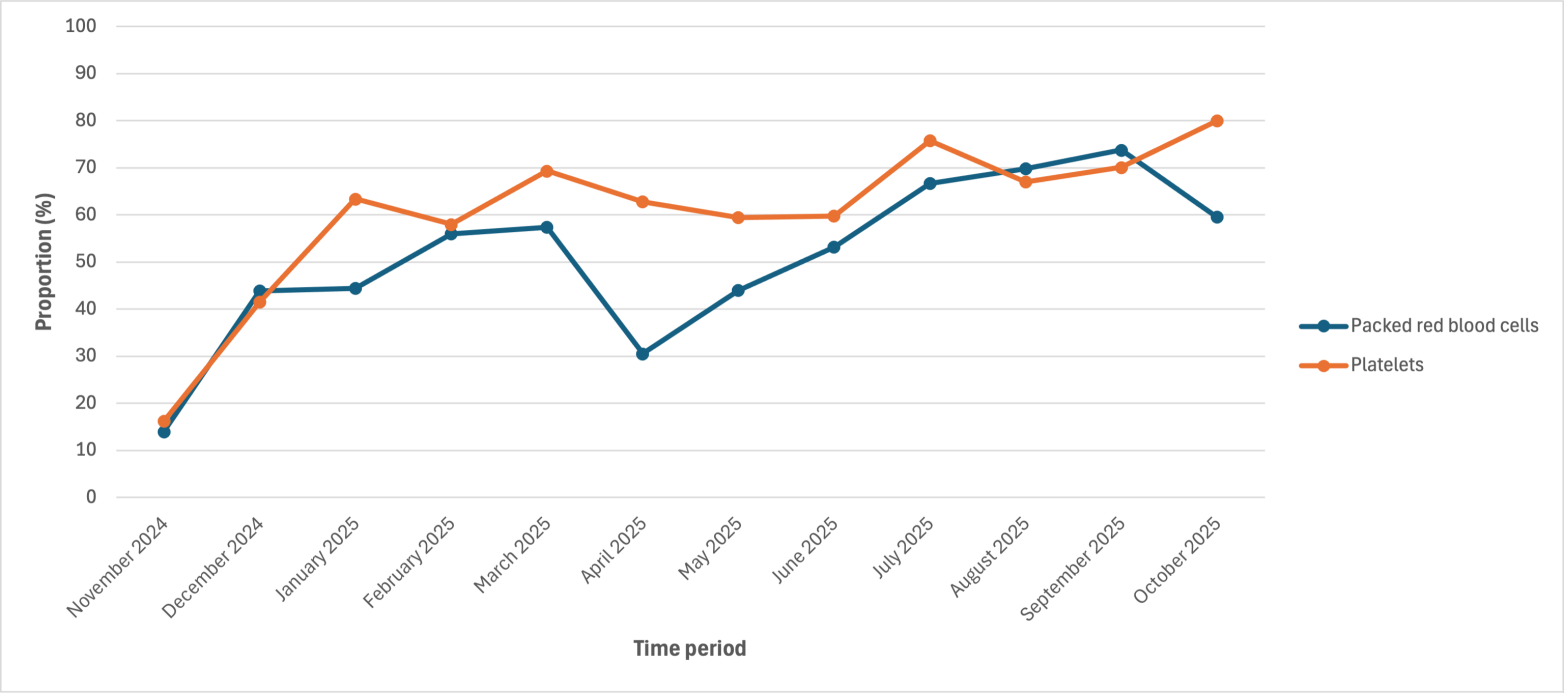
Proportion of blood product orders originating from a transfusion therapy plan.

### Impact on Efficiency

Changes in efficiency metrics preimplementation and postimplementation of the transfusion therapy plans are shown in Table 2. There was a significant difference in the timing between the transfusion and premedication orders (median 14, IQR 0-159 vs 10, IQR 0-145 min; *P*=.04). In the postimplementation period, there was a significant difference in the median time between the CBC being reported and the transfusion being released (178, IQR 35-394 vs 129, IQR 15-315 min; *P*<.001) and between the CBC being reported and transfusion administration (227, IQR 92-443 vs 181, IQR 76-367 min; *P<.*001) when using the therapy plan compared to the order set (Table 3). In the postintervention period, 1453 transfusions from therapy plans were released between 7 PM and 7 AM, suggesting an estimated avoidance of 4 overnight orders per night by the overnight resident or fellow.

**Table 2.** Impact of transfusion therapy plan implementation on efficiency.

Efficiency metric	Preimplementation cohort (n=2678 transfusions)	Postimplementation cohort (n=2777 transfusions)	Difference (95% CI)	*P* value
Type and screen drawn more than 5 minutes after CBC^a^^b^, % (n/N)	26 (271/1041)	29 (277/941)	3.4 (0.5 to 7.4)	.10
Time between CBC result and nurse releasing the transfusion orders (min), median (IQR)	143 (25-345)	146 (21-352)	2.5 (−6.2 to 11.2)	.58
Time between CBC result and transfusion administration (min), median (IQR)	201 (88-388)	198 (83-402)	−3.2 (−11.9 to 5.6)	.47
Time between transfusion order and premedication order for all patients receiving premedications (min), median (IQR)^c^	14 (0‐159)	10 (0‐145)	−5.8 (−21.6 to −3.8)	.04

a For packed red blood cell transfusions only (n=1041 preimplementation and n=941 postimplementation).

b CBC: complete blood count.

c For transfusions where a premedication was administered (n=422 preimplementation and n=399 postimplementation).

**Table 3.** Postimplementation efficiency and safety metrics stratified by transfusion ordering tool.

Metric	Order set (n=1143 transfusions)	Therapy plan (n=1634 transfusions)	Difference (95% CI)	*P* value
Efficiency metrics				
Type and screen drawn more than 5 minutes after CBC^a^^b^, % (n/N)	28 (129/468)	31 (148/473)	3.7 (2.1 to 9.5)	.24
Time between CBC result and nurse releasing the transfusion orders (min), median (IQR)	178 (35-394)	129 (15-315)	−42.9 (−56.9 to −29.4)	<.001
Time between CBC result and transfusion administration (min), median (IQR)	227 (92‐443)	181 (76‐367)	−37.5 (−51.1 to −24.1)	<.001
Time between blood order and premedication order for all patients receiving premedications (min), median (IQR)^c^	6 (0‐154)	10 (0‐127)	7.3 (−24.0 to 51.1)	.37
Safety metrics				
Premedications ordered for patient with blood product allergy^d^, % (n/N)	49 (110/224)	43 (182/427)	−6.4 (−14.5 to 1.6)	.13
Blood cell parameter prior to transfusion, mean (SD)				
Hemoglobin prior to pRBC^e^ transfusion (g/dL)	73 (11)	70 (5)	−2.5 (−3.6 to −1.4)	<.001
Platelet prior to platelet transfusion (kcells/µL)	24 (17)	19 (13)	−5.1 (−6.4 to −3.6)	<.001
Blood product volume was guideline concordant, % (n/N)				
pRBCs	6 (295/468)	85 (402/473)	22.0 (16.2 to 27.8)	<.001
Platelets	86 (579/675)	93 (1082/1161)	7.4 (4.4 to 10.4)	<.001
pRBCs and platelets	76 (874/1143)	91 (1484/1634)	14.3 (11.3 to 17.3)	<.001
Blood product was ordered as irradiated, % (n/N)				
pRBCs	68 (320/468)	97 (459/473)	28.6 (24.5 to 32.7)	<.001
Platelets	73 (494/675)	98 (1139/1161)	24.9 (21.7 to 28.1)	<.001
pRBCs and platelets	71 (814/1143)	98 (1598/1634)	26.6 (23.9 to 29.3)	<.001

a For packed red blood cell transfusions only (n=468 order set and n=473 therapy plan).

b CBC: complete blood count.

c For transfusions where a premedication was administered (n=171 order set and n=228 therapy plan).

d For patients with a platelet allergy receiving platelets, and patients with a red blood cell allergy receiving red blood cells only (n=110 order set and n=182 therapy plan).

e pRBC: packed red blood cell.

### Impact on Safety

The impact of the transfusion therapy plans on safety metrics is shown in Table 4. There was a significant difference in the proportion of patients with allergies who were ordered premedications (273/724, 38% vs 292/651, 45%; *P*=.008). No significant differences were identified in pretransfusion thresholds between the preimplementation and postimplementation cohorts. There was a significant increase in the proportion of patients receiving guideline-concordant transfusion volumes in the postimplementation period compared to the preimplementation period (58% vs 85%; *P<.*001). In the postimplementation period, there was a significant difference in the mean hemoglobin value prior to pRBC transfusion (73, SD 11 g/dL vs 70, SD 5 g/dL; *P*<.001), the mean platelet value prior to platelet transfusion (24, SD 17 kcells/µL vs 19, SD 13 kcells/µL; *P<.*001), the proportion of blood products where the volume ordered was guideline-concordant *(*87/1143, 76% vs 1484/1634, 91%; *P<.*001) and the proportion of blood products that were ordered as irradiated (814/1143, 71% vs 1595/1634, 98%; *P<.*001; Table 3).

**Table 4.** Impact of transfusion therapy plan implementation on transfusion safety.

Safety metric	Preimplementation cohort (n=2678 transfusions)	Postimplementation cohort (n=2777 transfusions)	Absolute difference (95% CI)	*P* value
Premedications ordered for patient with blood product allergy^a^, % (n/N)	38 (273/724)	45 (292/651)	7.2 (12.0 to 2.2)	.008
Blood cell parameter prior to transfusion, mean (SD)				
Hemoglobin prior to pRBC^b^ transfusion (g/dL)	71 (9)	71 (9)	−0.5 (−1.3 to 0.30)	.22
Platelet prior to platelet transfusion (kcells/µL)	21 (14)	20 (15)	−1.1 (−2.0 to -0.1)	.03
Blood product volume was guideline concordant, % (n/N)				
pRBCs	56 (583/1041)	74 (697/941)	18.1 (14.0 to 22.2)	<.001
Platelets	59 (960/1637)	90 (1661/1836)	31.9 (29.0 to 34.8)	<.001
pRBCs and platelets	58 (1543/2678)	85 (2358/2777)	27.3 (24.9 to 29.7)	<.001
Blood product was ordered as irradiated, % (n/N)				
pRBCs	81 (843/1041)	83 (779/941)	1.8 (−1.4 to 5.0)	.30
Platelets	86 (1400/1637)	89 (1633/1836)	3.4 (1.2 to 5.6)	<.01
pRBCs and platelets	84 (2243/2678)	87 (2412/2777)	3.0 (1.1 to 4.9)	.003

a For patients with a platelet allergy receiving platelets, and patients with a red blood cell allergy receiving red blood cells only (n=273 preimplementation and n=292 postimplementation)

b pRBC: packed red blood cell.

### Health Care Practitioner Experiences

**Figure 4.**
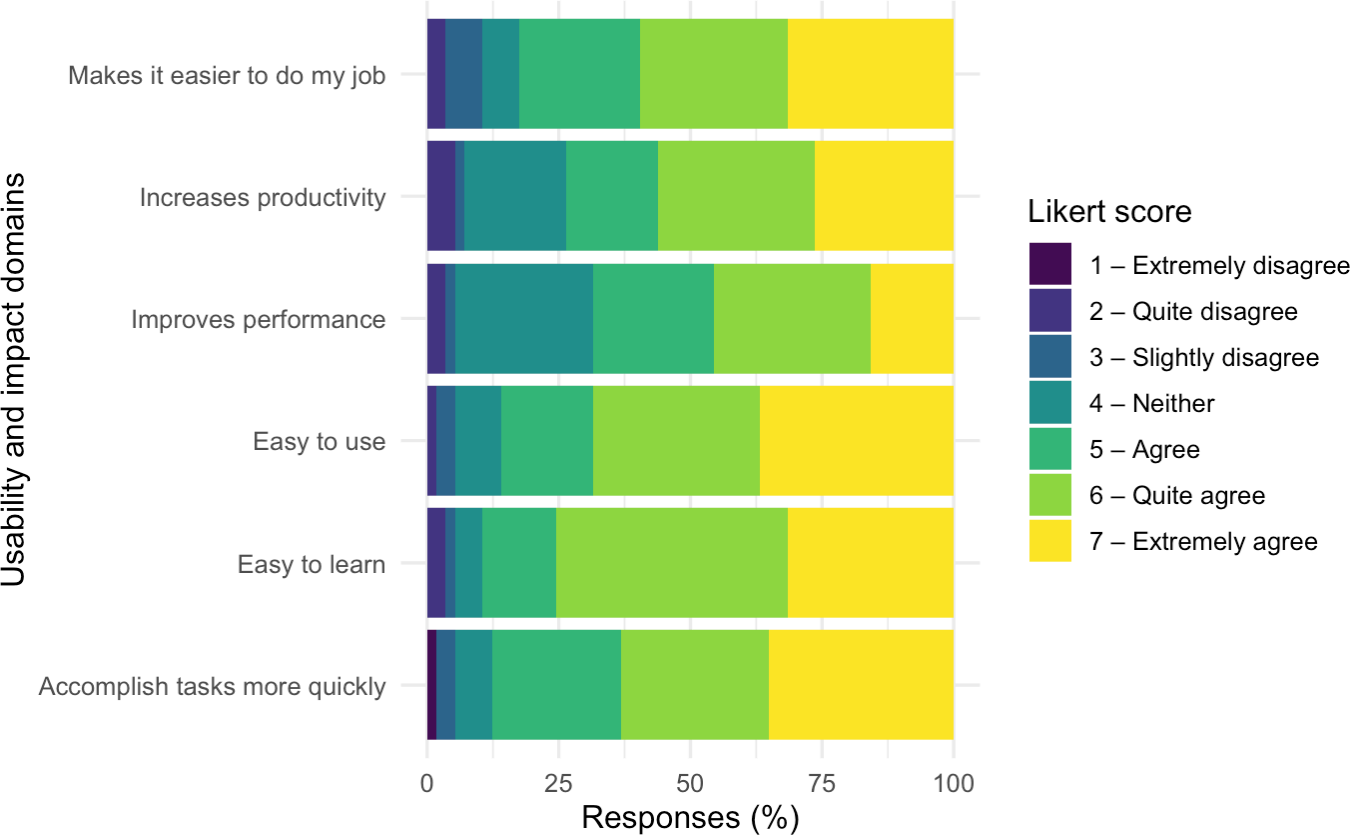
Participant ratings of the usability and impact of the transfusion therapy plans.

The health care practitioner experience survey was completed by 57 individuals (27 nurses and 30 providers). This represents a response rate of approximately 15% of our oncology workforce. Of the 27 nurses, 19 worked primarily in the inpatient setting, and 8 worked primarily in the outpatient setting. Of the 30 providers, 4 were attending physicians, 8 were nurse practitioners or physician assistants, 9 were hematology or oncology fellows, and 9 were general pediatrics residents. Results of the abbreviated TAM questionnaire are presented in Figure 4 and Table S3 in Multimedia Appendix 1. Self-reported preferences related to ordering tools for blood products are shown in Table 5. Overall, 95% (n=54) of respondents felt we should continue to use the transfusion therapy plans.

**Table 5.** Preferences of oncology clinicians regarding ordering tools for transfusions.

Questions	Transfusion order set preferred, n (%)	No difference, n (%)	Transfusion therapy plan preferred, n (%)
Oncology provider-specific questions (n=21)			
Which tool allows you to order blood more efficiently?	2 (9.5)	0 (0)	19 (90.5)
Which tool allows you to order blood in the safest way?	3 (14.3)	16 (76.2)	2 (9.5)
Which tool best ensures your patients’ blood is ordered with the correct precautions (eg, irradiated)?	2 (9.5)	16 (76.2)	3 (14.3)
Which tool best identifies a patient’s specific transfusion parameters?	2 (9.5)	15 (70.5)	4 (19)
Which tool best ensures that your patients have the correct blood product volume ordered?	1 (4.8)	16 (76.2)	4 (19)
Which tool best ensures that your patients have the correct pre-medications ordered?	2 (9.5)	18 (85.7)	1 (4.8)
Trainee-specific question (n=18)			
Which tool results in less unnecessary overnight pages?	0 (0)	12 (66.7)	6 (33.3)
Nursing-specific questions (n=27)			
Which tool allows you to access blood orders most easily?	0 (0)	22 (81.5)	5 (18.5)
Which tools allows you to administer blood in the safest way?	3 (11.1)	14 (51.9)	10 (37)
Which tool results in the fewest clicks or least amount of time in Epic?	8 (29.6)	16 (59.6)	3 (11.1)
Which tool resulted in the clearest communication with ordering providers?	8 (29.6)	17 (63.3)	2 (7.4)
Which tool results in fewer delays in care?	3 (11.1)	21 (77.8)	3 (11.1)

## Discussion

In this study, we evaluated the implementation of transfusion therapy plans for pediatric oncology and HSCT patients and demonstrated meaningful improvements in efficiency, safety, and health care practitioner experience compared to traditional transfusion order sets. Collectively, these findings suggest that reusable transfusion therapy plans represent a valuable EHR-based intervention for supporting complex, high-risk ordering workflows in pediatric cancer care.

Although only one-quarter of eligible patients had a transfusion therapy plan applied during the evaluation period, over half of the patients who required a transfusion had a therapy plan, and nearly 60% of all transfusion orders originated from a therapy plan. Importantly, adoption increased over time. This is consistent with prior EHR-based workflow interventions, where uptake is often gradual and influenced by clinician familiarity, trust in the tool, and perceived value [19]. The higher adoption rate among patients who actually received transfusions suggests that clinicians may preferentially apply therapy plans to patients with anticipated or recurrent transfusion needs, aligning with the intended use case of the tool. Adoption rates also differed by section, with patients with leukemia or lymphoma having a much lower adoption rate. This is understandable, given that many patients with leukemia have 2 to 3 years of maintenance therapy where transfusions are infrequent and a transfusion therapy plan might not be useful.

Use of transfusion therapy plans was associated with improvements in several measures of operational efficiency. In the postimplementation cohort, we demonstrated an increase in premedication usage and improved timing of ordering for outpatients with documented allergies; however, given these were modest improvements, these changes may not have significant clinical impacts. Additionally, we saw no improvement in the time between the CBC being resulted and the transfusion being released or administered in the precohort versus postcohort. In contrast, we did see a difference in the postcohort when comparing the order set to the therapy plan. This likely reflects that in the postcohort, given the significant usage of the order set, the impact of the improved timeliness of blood administration with the therapy plan was ameliorated when analyzing the entire cohort instead of conducting a subanalysis by ordering tool. The effect size of these changes in the postimplementation period between the 2 ordering tools was clinically meaningful, with blood being administered 37 minutes earlier with the use of the therapy plan.

We observed significant improvements in guideline-concordant transfusion volume ordering following the implementation of the therapy plans. This finding is particularly notable given our prior work demonstrating that adherence to care pathway recommendations for supportive care in pediatric cancer is often low [20]. Ensuring that transfusion volumes are guideline-consistent is important as inappropriate transfusion volumes are a common source of safety events [2,3,6]. By embedding hard stops and weight-based dosing logic directly into the order configuration, the therapy plans effectively operationalized institutional transfusion policies at the point of order entry. The higher rates of irradiated blood product ordering in the postimplementation period further support the role of structured, reusable orders in reducing reliance on clinician memory for critical safety requirements in immunocompromised populations. These changes are likely to have a meaningful clinical impact, given the magnitude of the improvements and the impact of guideline-concordant care on clinical outcomes.

Health care practitioner feedback strongly supported the continued use of transfusion therapy plans, with high ratings for perceived usefulness and ease of use across both nursing and provider roles. High acceptability across disciplines is especially important in transfusion workflows, which require close coordination between providers, nurses, and the blood bank. Ensuring usability is particularly important given the known impact of EHR usage on clinician burnout [21-23]. When clinicians were asked about their preferences between the order set and therapy plan, the majority found no difference between the tools but supported the continued use of the transfusion therapy plans.

This study has several limitations. First, as a single-center observational study, findings may not be generalizable to institutions with different EHR configurations, transfusion policies, or staffing models. However, the concept of using transfusion therapy plans instead of ad hoc orders would be easily transferable to other centers. Second, the response rate to our survey was low and may not represent the opinions of all staff. Third, adoption was incomplete during the study period, and outcomes may further improve with sustained use and targeted education. Future work should explore strategies to increase adoption, including clinical decision support to suggest therapy plan application, and expansion to other patient populations that receive high volumes of transfusions. Multicenter evaluations will be important to assess generalizability as well.

In summary, our findings suggest that transfusion therapy plans are a promising approach to improving the safety, efficiency, and user experience of blood product ordering in pediatric oncology and HSCT populations.

## Supplementary material

10.2196/93346 Multimedia Appendix 1 Comparison of transfusion order sets and transfusion therapy plan workflows.

10.2196/93346 Multimedia Appendix 2 Transfusion therapy plan configuration—location of the packed red blood cell and platelet transfusion therapy plans within the treatment activity.

10.2196/93346 Multimedia Appendix 3 Transfusion therapy plan configuration—packed red blood cell transfusion therapy plan showing predefined dosing options, transfusion thresholds, and optional premedications.

10.2196/93346 Multimedia Appendix 4 Transfusion therapy plan configuration—platelet transfusion therapy plan showing weight-based dosing constraints, transfusion thresholds, and emergency medications.

## Abbreviations

BMT: bone marrow transplant
CBC: complete blood count
EHR: electronic health record
HSCT: hematopoietic stem cell transplant
pRBC: packed red blood cell
REDCap: Research Electronic Data Capture
TAM: technology acceptance model
