# Supplementary material for: Implementation and Evaluation of an Alternative Electronic Health Record Tool for Ordering Blood Products in Pediatric Oncology and Stem Cell Transplantation: Mixed Methods Analysis
Source: JMIR Med Inform. 2026 May 15;14:e93346. doi: 10.2196/93346 (PMC13178816; doi:10.2196/93346)
Supplement: Multimedia Appendix 1 [file medinform-v14-e93346-s001.docx]

**Supplemental Table 1: Comparison of Transfusion Order Sets and Transfusion Therapy Plan Workflows:**

|  | **Transfusion Order Set** | **Transfusion Therapy Plan** |
| --- | --- | --- |
| **Blood Product Ordering** | When a transfusion is needed, the appropriate transfusion order set (packed red blood cells or platelets) is entered by a provider.   Orders must be entered each time a transfusion is required. | Red blood cell orders, and/or platelet orders are entered by a provider in advance of an identified need for a transfusion or with first transfusion. The therapy plan has a pre-specified parameter in terms of hemoglobin or platelet threshold for when to use.   Orders only need to be entered once, and can then be re-used for subsequent transfusions. |
| **Nursing Review of Orders** | When a transfusion is needed, the bedside nurse reviews the entered transfusion orders.  If the nurse agrees that a transfusion is required, the nurse releases all of the entered orders. | When a transfusion is needed, the bedside nurse reviews the entered transfusion orders.   If the nurse agrees that a transfusion is required, and the patient’s hemoglobin or platelets is below the threshold specified in the therapy plan, the nurse releases all of the entered orders. If the hemoglobin or platelets is not below the specified threshold, the nurse requests a communication order from the provider indicating that the therapy plan should be used. |
|  | Once the nurse releases the orders, they are now active orders that can be used. A “prepare” order is sent to the blood bank. | |
| **Blood Bank Preparation of Blood Product** | Blood bank receives a “prepare” order from the orders released by the nurse indicating what blood product to prepare along with the required volume and precautions.   Blood bank prepares the required blood product and sends the product to the unit/clinic where transfusion will occur. | |
| **Transfusion of Blood Product** | The nurse administers any required pre-medications, the blood product (via a transfuse order), monitors for transfusion reactions, and administers emergency medications if required. | |

**Supplemental Table 2: Transfusion Therapy Plan Usage Stratified by Clinical Disease Group**

|  | Leukemia & Lymphoma | Solid Tumor | Neuro Oncology | Bone Marrow Transplant |
| --- | --- | --- | --- | --- |
| Proportion of Eligible Patients with any Transfusion Therapy Plan*: | 32% (n=97/307) | 86% (n=92/107) | 100% (n=75/75) | 100% (n=95/95) |
| Proportion of Transfused Patients with any Transfusion Therapy Plan*: | 47%  (n=45/95) | 93% (n=62/67) | 100% (n=69/69) | 100% (n=95/95) |

*Packed red blood cell or platelet transfusion therapy plan

**Supplemental Table 3: Participant Rating of Usability and Impact of Transfusion Therapy Plans**

|  | **Extremely Disagree** | **Quite Disagree** | **Slightly Disagree** | **Neither** | **Agree** | **Quite Agree** | **Extremely Agree** |
| --- | --- | --- | --- | --- | --- | --- | --- |
| **Accomplish tasks more quickly** | 2% (n=1) | 0% (n=0) | 4% (n=2) | 7% (n=4) | 25% (n=14) | 28% (n=16) | 35 (n=20) |
| **Easier to do job** | 0% (n=0) | 4% (n=2) | 7% (n=4) | 7% (n=4) | 23% (n=13) | 28% (n=16) | 32% (n=18) |
| **Easy to learn** | 0% (n=0) | 4% (n=2) | 2% (n=1) | 5% (n=3)_ | 14% (n=8) | 44% (n=25) | 32% (n=18) |
| **Easy to use** | 0% (n=0) | 2% (n=1) | 4% (n=2) | 9% (n=5) | 18% (n=10) | 32% (n=18) | 37% (n=21) |
| **Improves performance** | 0% (n=0) | 4% (n=2) | 2% (n=1) | 26% (n=15) | 23% (n=13) | 30% (n=17) | 16% (n=9) |
| **Increases productivity** | 0% (n=0) | 5% (n=3) | 2% (n=1) | 20% (n=11) | 18% (n=10) | 30% (n=17) | 26% (n=15) |
