# Supplementary material for: Implementation and Evaluation of an Alternative Electronic Health Record Tool for Ordering Blood Products in Pediatric Oncology and Stem Cell Transplantation: Mixed Methods Analysis
Source: JMIR Med Inform. 2026 May 15;14:e93346. doi: 10.2196/93346 (PMC13178816; doi:10.2196/93346)
Supplement: Multimedia Appendix 2 [file medinform-v14-e93346-s002.docx]

**Multimedia Appendix 1: Transfusion Therapy Plan Configuration- Location of the packed red blood cell and platelet transfusion therapy plans within the Treatment activity.


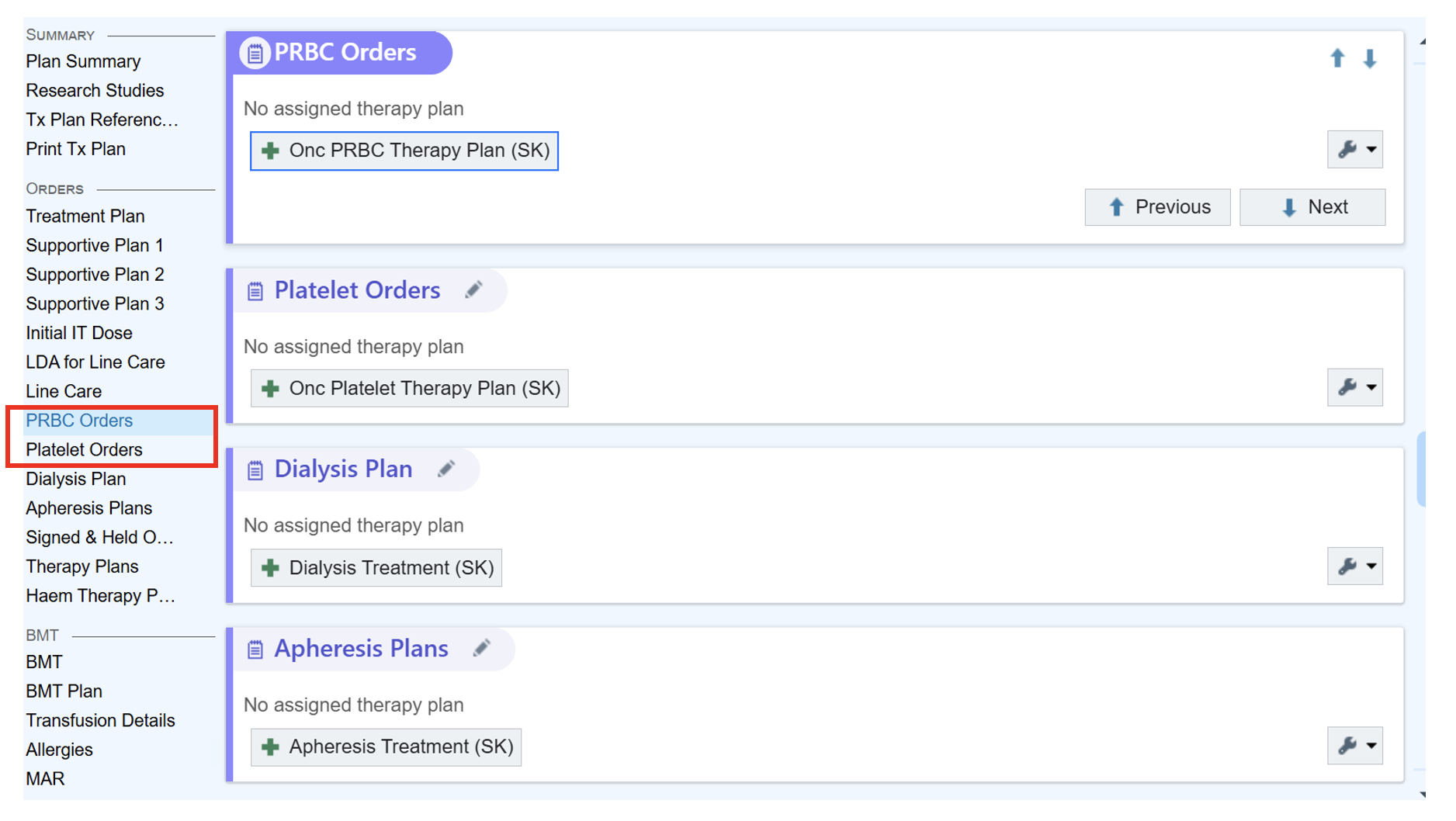
**
