# Supplementary material for: Implementation and Evaluation of an Alternative Electronic Health Record Tool for Ordering Blood Products in Pediatric Oncology and Stem Cell Transplantation: Mixed Methods Analysis
Source: JMIR Med Inform. 2026 May 15;14:e93346. doi: 10.2196/93346 (PMC13178816; doi:10.2196/93346)
Supplement: Multimedia Appendix 4 [file medinform-v14-e93346-s004.docx]

**Multimedia Appendix 3: Transfusion Therapy Plan Configuration- Platelet transfusion therapy plan showing weight-based dosing constraints, transfusion thresholds, and emergency medications.

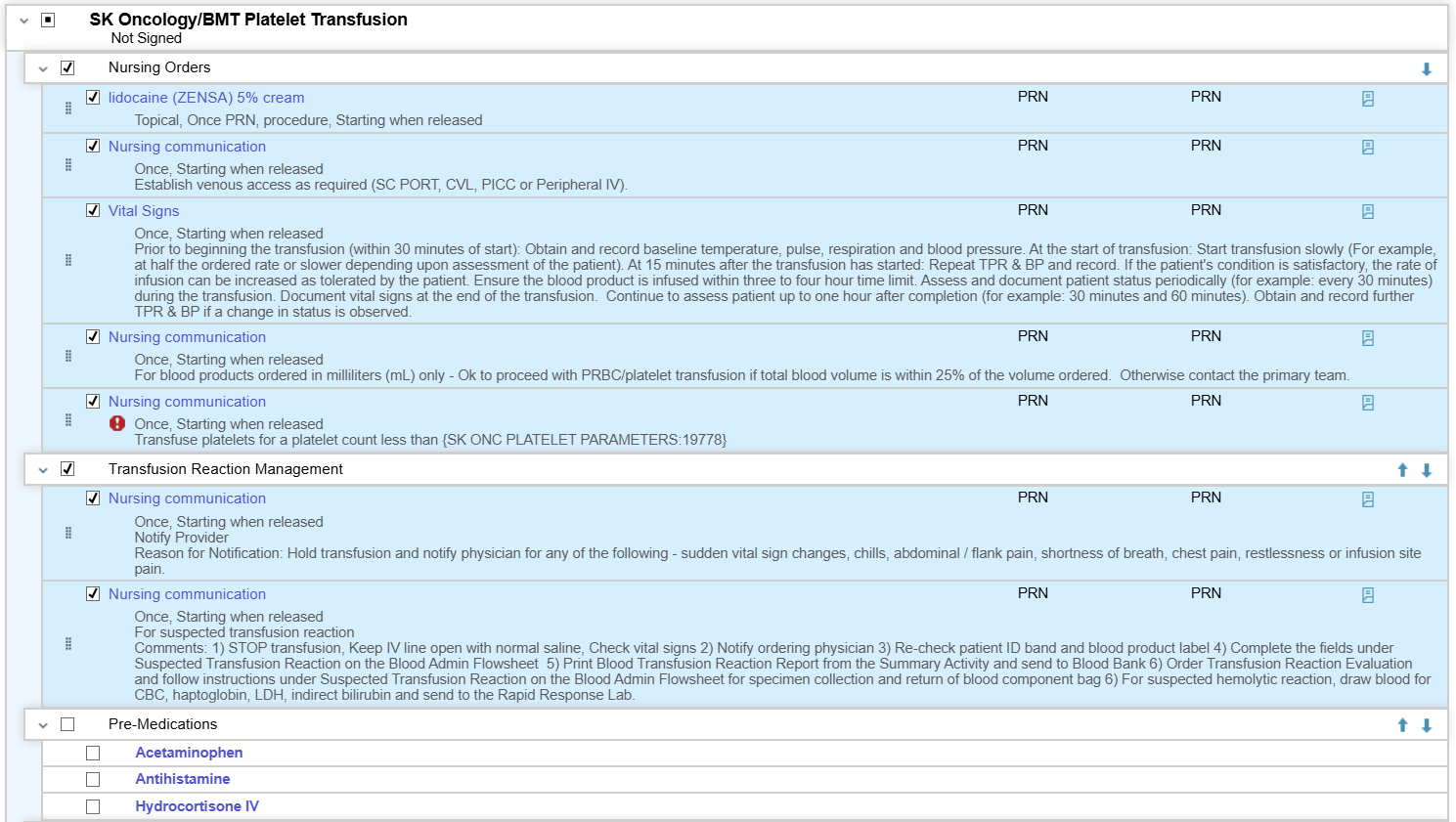


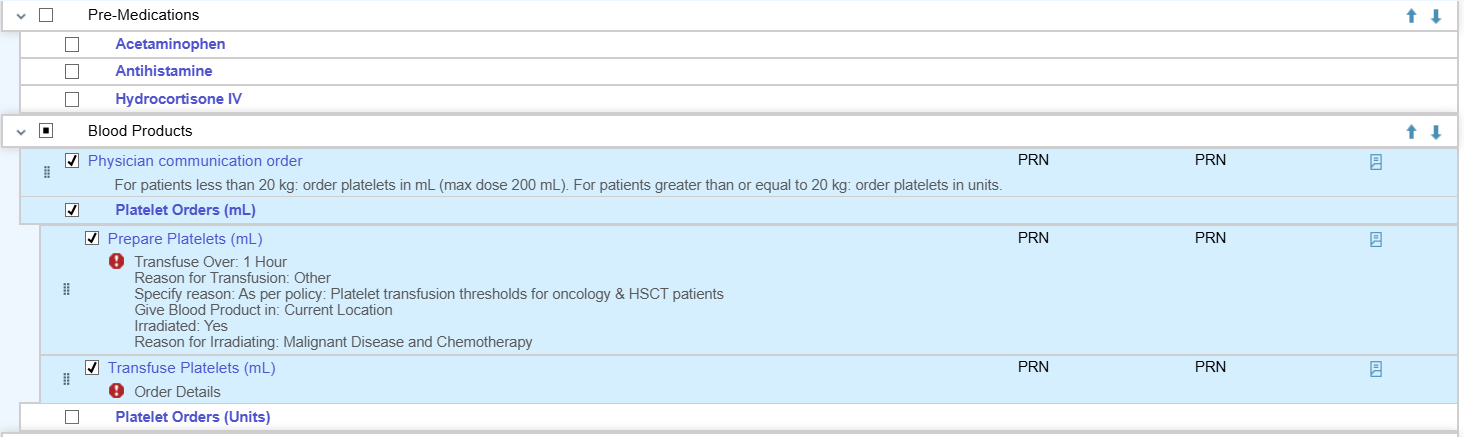


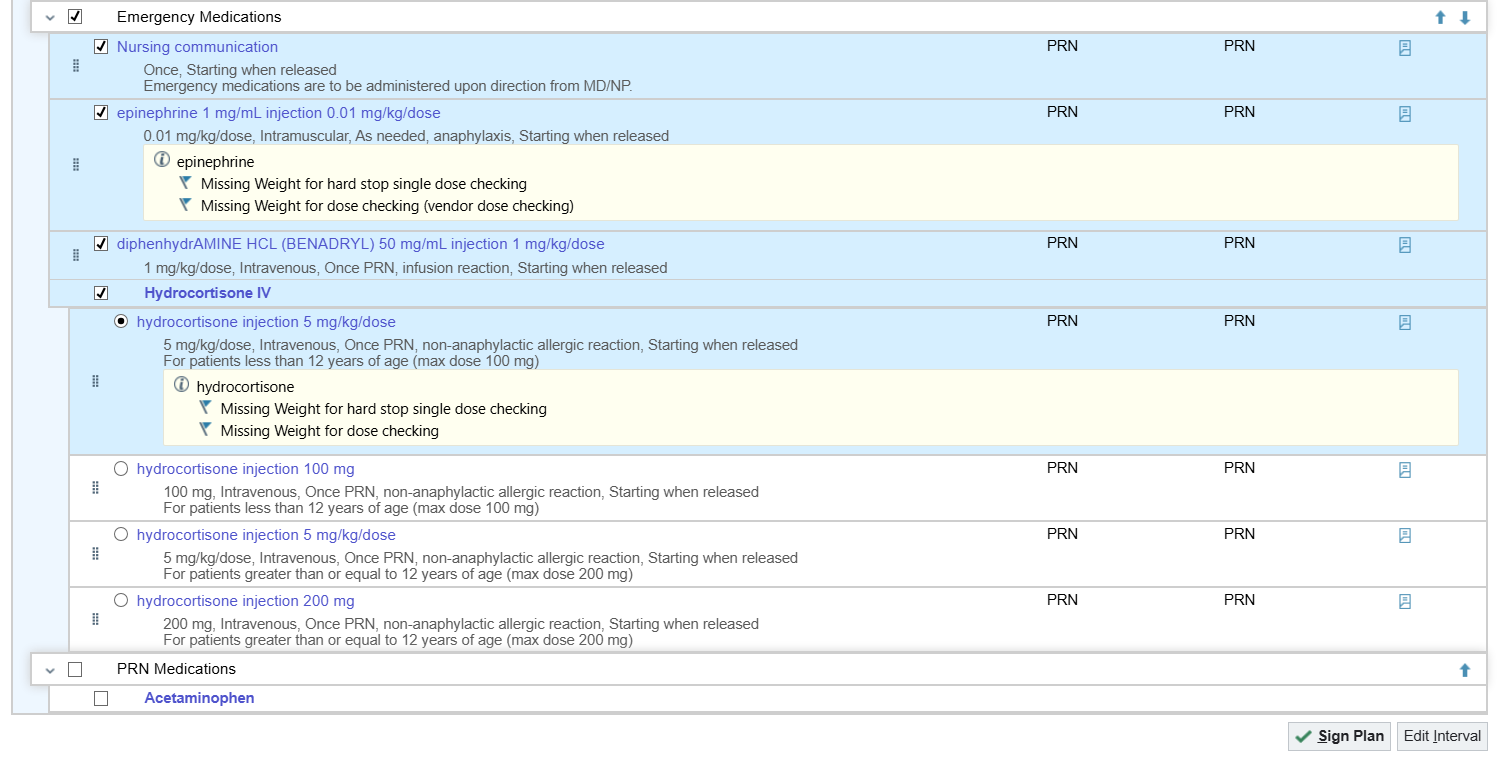
**
